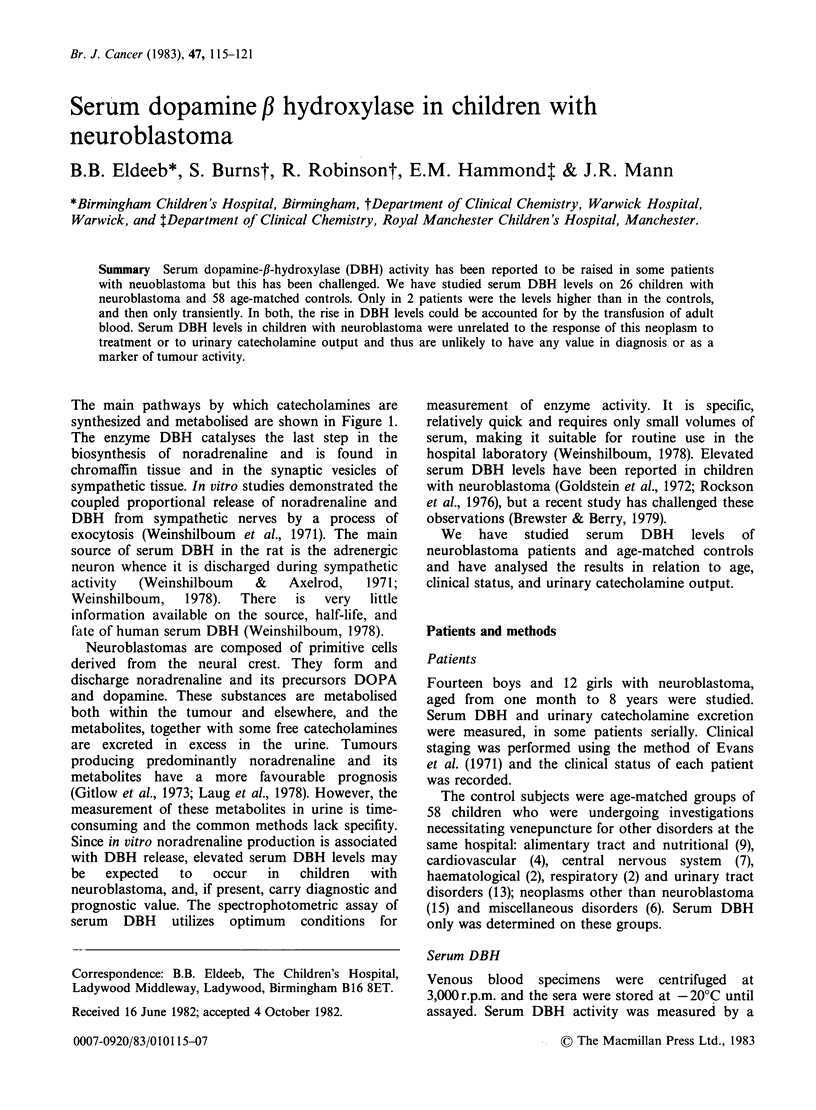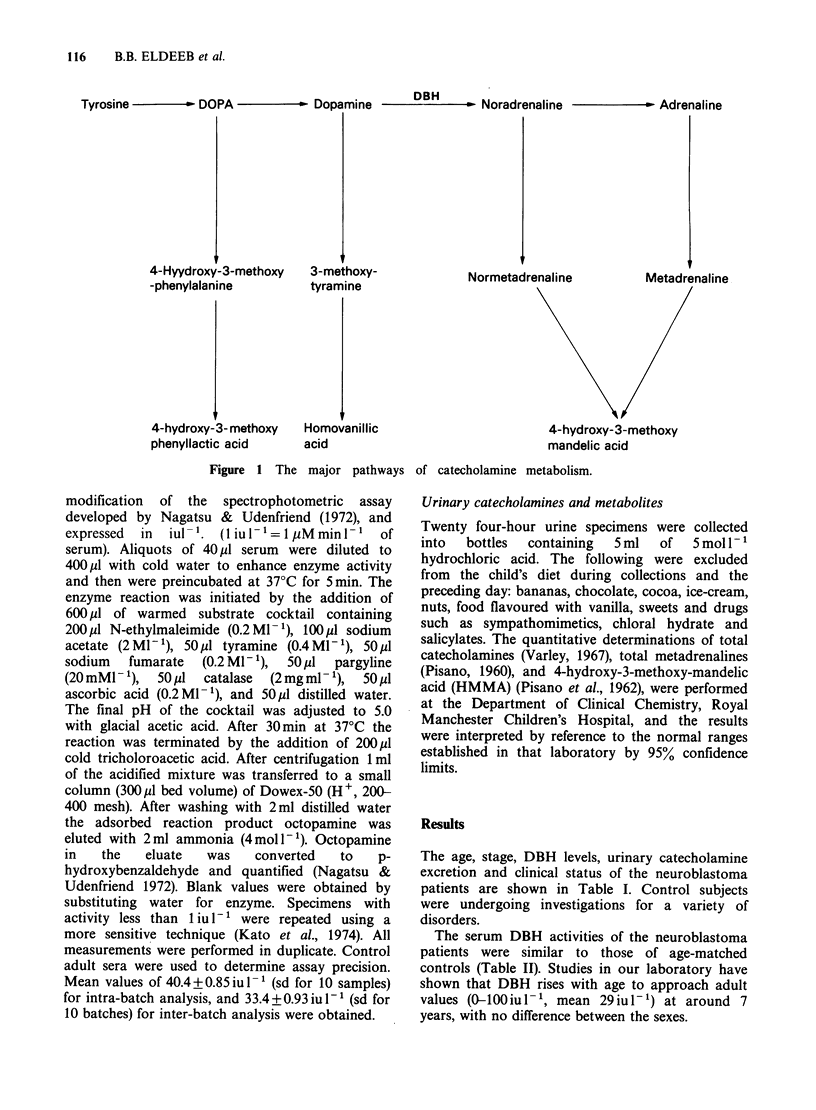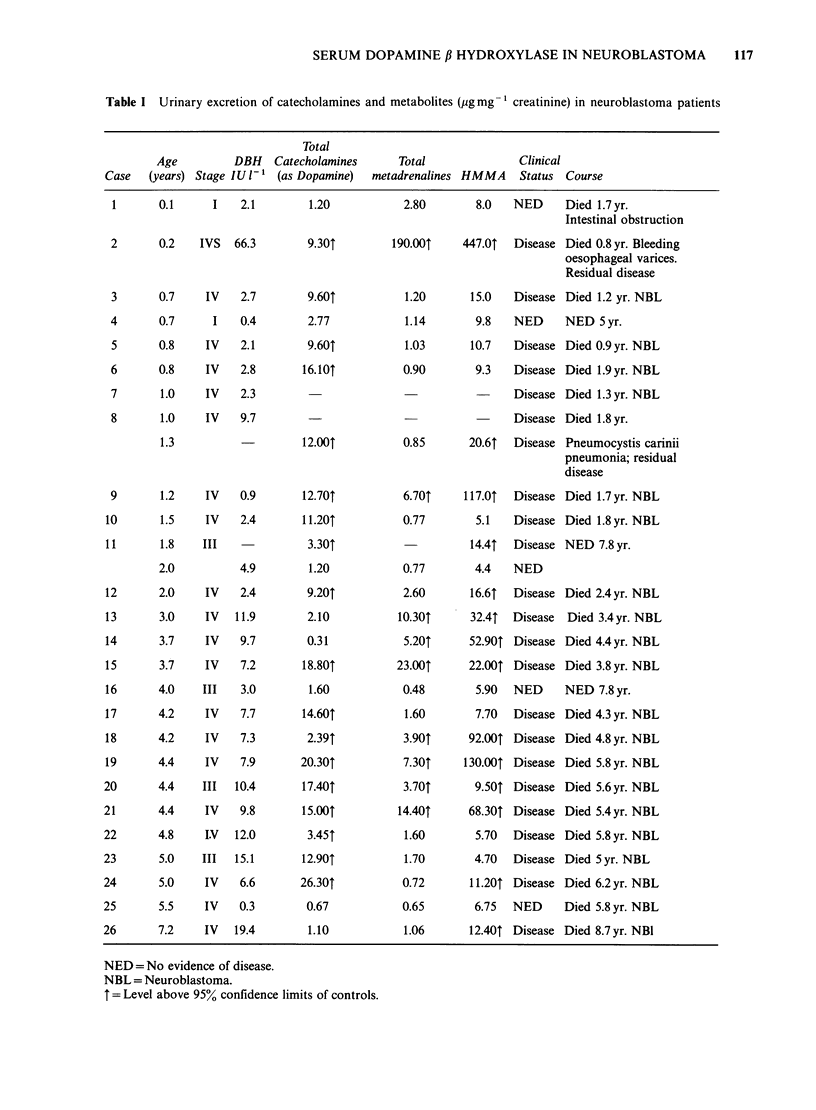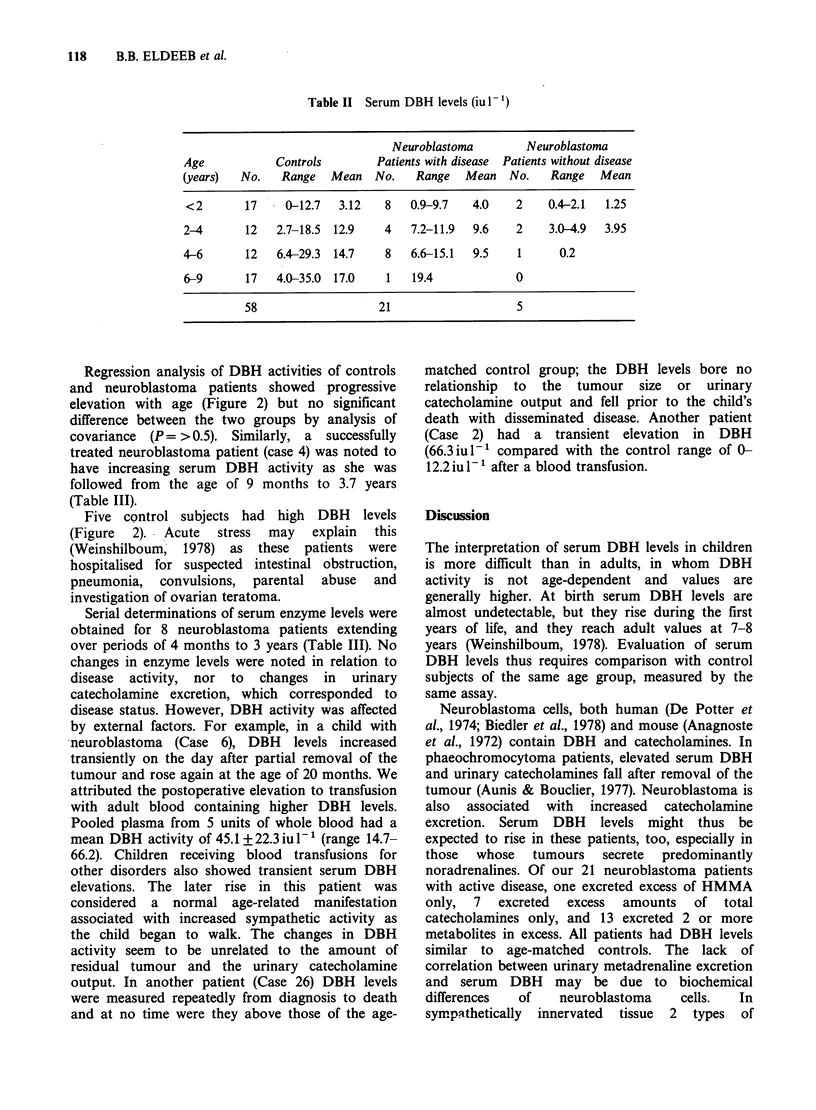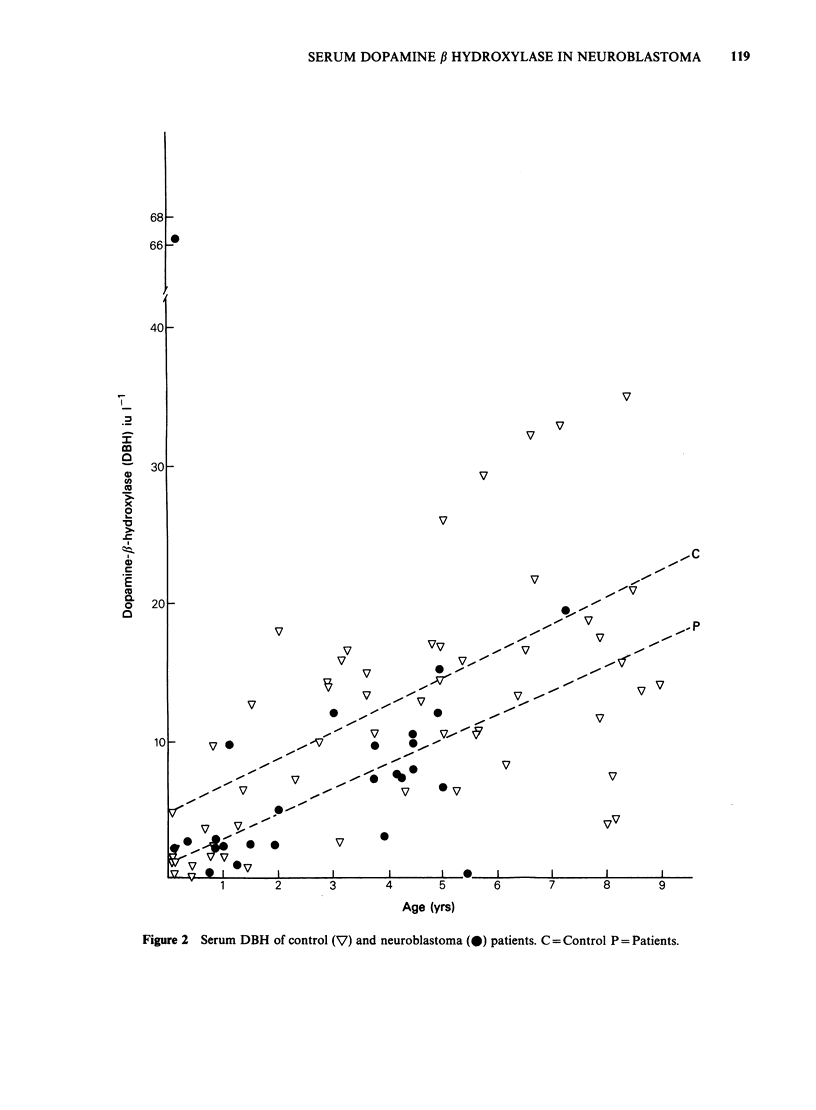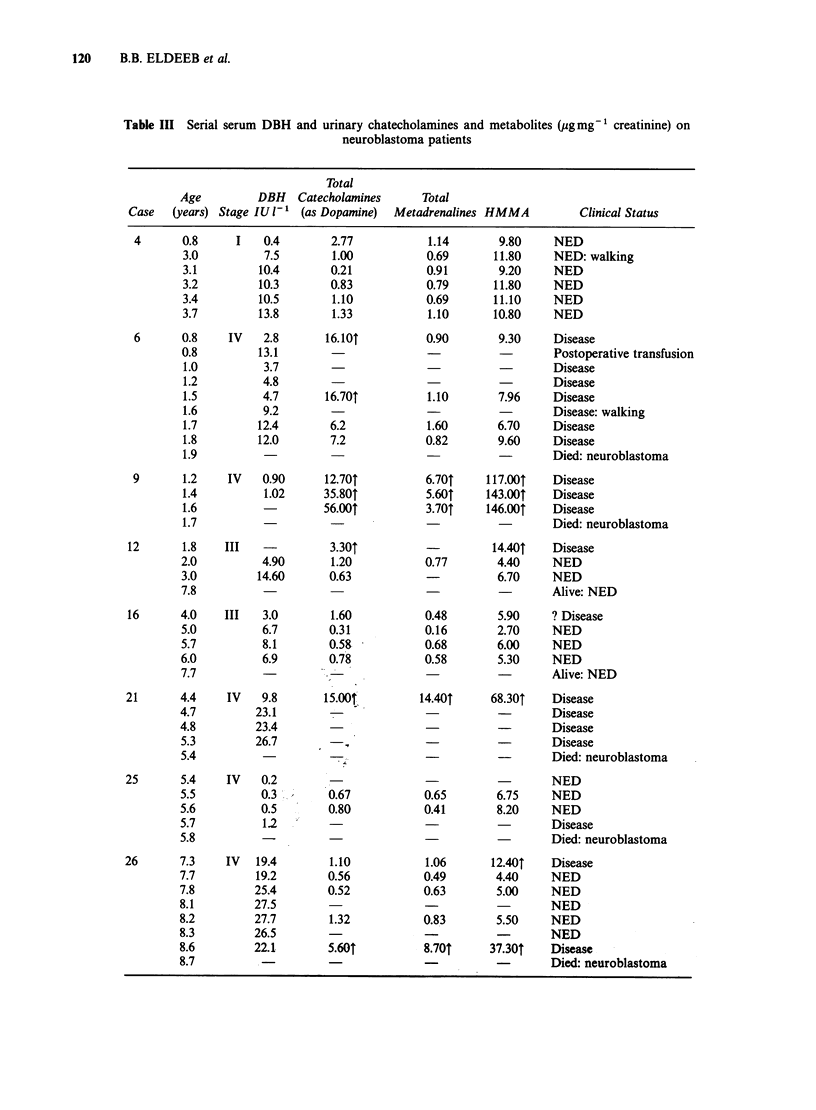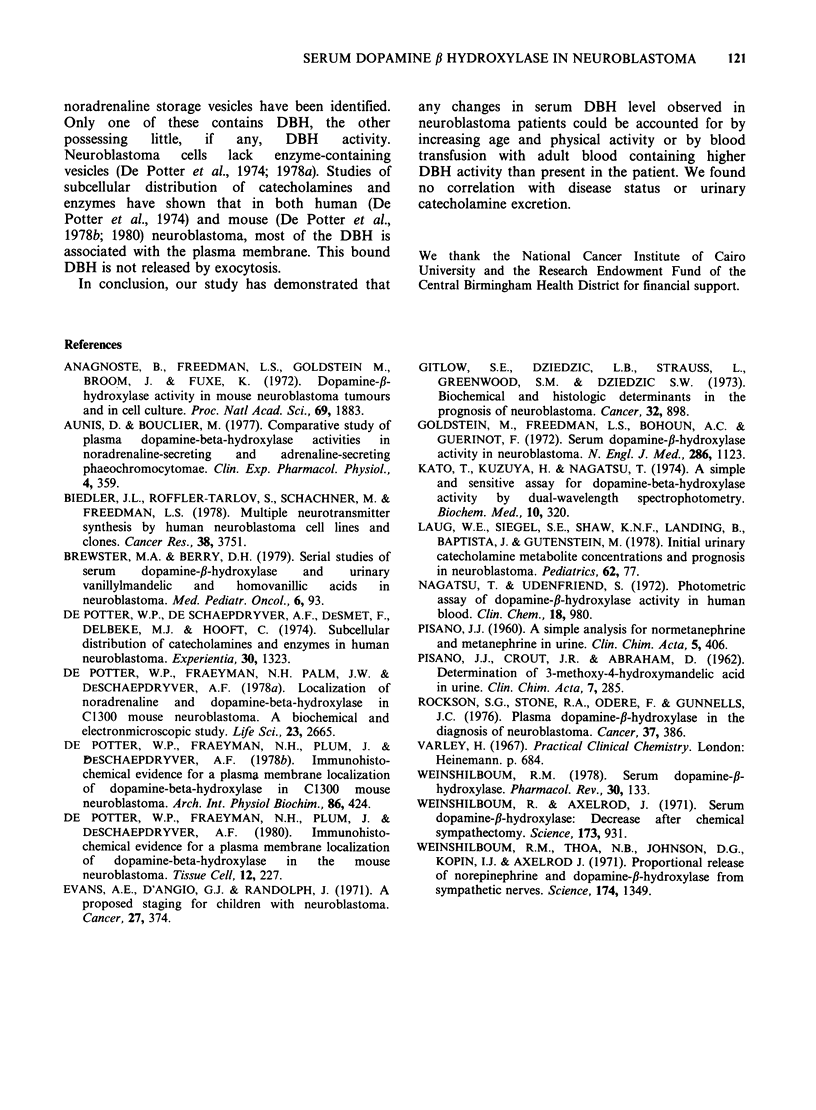# Serum dopamine beta hydroxylase in children with neuroblastoma.

**DOI:** 10.1038/bjc.1983.14

**Published:** 1983-01

**Authors:** B. B. Eldeeb, S. Burns, R. Robinson, E. M. Hammond, J. R. Mann

## Abstract

Serum dopamine-beta-hydroxylase (DBH) activity has been reported to be raised in some patients with neuoblastoma but this has been challenged. We have studied serum DBH levels on 26 children with neuroblastoma and 58 age-matched controls. Only in 2 patients were the levels higher than in the controls, and then only transiently. In both, the rise in DBH levels could be accounted for by the transfusion of adult blood. Serum DBH levels in children with neuroblastoma were unrelated to the response of this neoplasm to treatment or to urinary catecholamine output and thus are unlikely to have any value in diagnosis or as a marker of tumour activity.


Br. J. Cancer (1983), **47**, 115–121

### Serum dopamine $\beta$ hydroxylase in children with neuroblastoma

B.B. Eldeeb*, S. Burns†, R. Robinson†, E.M. Hammond‡ & J.R. Mann

**Birmingham Children's Hospital, Birmingham, †Department of Clinical Chemistry, Warwick Hospital, Warwick, and ‡Department of Clinical Chemistry, Royal Manchester Children's Hospital, Manchester.*

**Summary** Serum dopamine-$\beta$-hydroxylase (DBH) activity has been reported to be raised in some patients with neuoblastoma but this has been challenged. We have studied serum DBH levels on 26 children with neuroblastoma and 58 age-matched controls. Only in 2 patients were the levels higher than in the controls, and then only transiently. In both, the rise in DBH levels could be accounted for by the transfusion of adult blood. Serum DBH levels in children with neuroblastoma were unrelated to the response of this neoplasm to treatment or to urinary catecholamine output and thus are unlikely to have any value in diagnosis or as a marker of tumour activity.

The main pathways by which catecholamines are synthesized and metabolised are shown in Figure 1. The enzyme DBH catalyses the last step in the biosynthesis of noradrenaline and is found in chromaffin tissue and in the synaptic vesicles of sympathetic tissue. *In vitro* studies demonstrated the coupled proportional release of noradrenaline and DBH from sympathetic nerves by a process of exocytosis (Weinshilboum *et al.*, 1971). The main source of serum DBH in the rat is the adrenergic neuron whence it is discharged during sympathetic activity (Weinshilboum & Axelrod, 1971; Weinshilboum, 1978). There is very little information available on the source, half-life, and fate of human serum DBH (Weinshilboum, 1978).

Neuroblastomas are composed of primitive cells derived from the neural crest. They form and discharge noradrenaline and its precursors DOPA and dopamine. These substances are metabolised both within the tumour and elsewhere, and the metabolites, together with some free catecholamines are excreted in excess in the urine. Tumours producing predominantly noradrenaline and its metabolites have a more favourable prognosis (Gitlow *et al.*, 1973; Laug *et al.*, 1978). However, the measurement of these metabolites in urine is time-consuming and the common methods lack specifity. Since *in vitro* noradrenaline production is associated with DBH release, elevated serum DBH levels may be expected to occur in children with neuroblastoma, and, if present, carry diagnostic and prognostic value. The spectrophotometric assay of serum DBH utilizes optimum conditions for measurement of enzyme activity. It is specific, relatively quick and requires only small volumes of serum, making it suitable for routine use in the hospital laboratory (Weinshilboum, 1978). Elevated serum DBH levels have been reported in children with neuroblastoma (Goldstein *et al.*, 1972; Rockson *et al.*, 1976), but a recent study has challenged these observations (Brewster & Berry, 1979).

We have studied serum DBH levels of neuroblastoma patients and age-matched controls and have analysed the results in relation to age, clinical status, and urinary catecholamine output.

### Patients and methods

### *Patients*

Fourteen boys and 12 girls with neuroblastoma, aged from one month to 8 years were studied. Serum DBH and urinary catecholamine excretion were measured, in some patients serially. Clinical staging was performed using the method of Evans *et al.* (1971) and the clinical status of each patient was recorded.

The control subjects were age-matched groups of 58 children who were undergoing investigations necessitating venepuncture for other disorders at the same hospital: alimentary tract and nutritional (9), cardiovascular (4), central nervous system (7), haematological (2), respiratory (2) and urinary tract disorders (13); neoplasms other than neuroblastoma (15) and miscellaneous disorders (6). Serum DBH only was determined on these groups.

### *Serum DBH*

Venous blood specimens were centrifuged at 3,000 r.p.m. and the sera were stored at $-20°C$ until assayed. Serum DBH activity was measured by a

Correspondence: B.B. Eldeeb, The Children's Hospital, Ladywood Middleway, Ladywood, Birmingham B16 8ET.

Received 16 June 1982; accepted 4 October 1982.

0007-0920/83/010115–07

© The Macmillan Press Ltd., 1983

**Figure 1** The major pathways of catecholamine metabolism.

modification of the spectrophotometric assay developed by Nagatsu & Udenfriend (1972), and expressed in $iul^{-1}$. ($1\,iu\,l^{-1} = 1\,\mu M\,min\,l^{-1}$ of serum). Aliquots of $40\,\mu l$ serum were diluted to $400\,\mu l$ with cold water to enhance enzyme activity and then were preincubated at 37°C for 5 min. The enzyme reaction was initiated by the addition of $600\,\mu l$ of warmed substrate cocktail containing $200\,\mu l$ N-ethylmaleimide ($0.2\,Ml^{-1}$), $100\,\mu l$ sodium acetate ($2\,Ml^{-1}$), $50\,\mu l$ tyramine ($0.4\,Ml^{-1}$), $50\,\mu l$ sodium fumarate ($0.2\,Ml^{-1}$), $50\,\mu l$ pargyline ($20\,mMl^{-1}$), $50\,\mu l$ catalase ($2\,mg\,ml^{-1}$), $50\,\mu l$ ascorbic acid ($0.2\,Ml^{-1}$), and $50\,\mu l$ distilled water. The final pH of the cocktail was adjusted to 5.0 with glacial acetic acid. After 30 min at 37°C the reaction was terminated by the addition of $200\,\mu l$ cold tricholoroacetic acid. After centrifugation 1 ml of the acidified mixture was transferred to a small column ($300\,\mu l$ bed volume) of Dowex-50 ($H^+$, 200–400 mesh). After washing with 2 ml distilled water the adsorbed reaction product octopamine was eluted with 2 ml ammonia ($4\,mol\,l^{-1}$). Octopamine in the eluate was converted to p-hydroxybenzaldehyde and quantified (Nagatsu & Udenfriend 1972). Blank values were obtained by substituting water for enzyme. Specimens with activity less than $1\,iu\,l^{-1}$ were repeated using a more sensitive technique (Kato *et al.*, 1974). All measurements were performed in duplicate. Control adult sera were used to determine assay precision. Mean values of $40.4 \pm 0.85\,iu\,l^{-1}$ (sd for 10 samples) for intra-batch analysis, and $33.4 \pm 0.93\,iu\,l^{-1}$ (sd for 10 batches) for inter-batch analysis were obtained.

*Urinary catecholamines and metabolites*

Twenty four-hour urine specimens were collected into bottles containing 5 ml of $5\,mol\,l^{-1}$ hydrochloric acid. The following were excluded from the child's diet during collections and the preceding day: bananas, chocolate, cocoa, ice-cream, nuts, food flavoured with vanilla, sweets and drugs such as sympathomimetics, chloral hydrate and salicylates. The quantitative determinations of total catecholamines (Varley, 1967), total metadrenalines (Pisano, 1960), and 4-hydroxy-3-methoxy-mandelic acid (HMMA) (Pisano *et al.*, 1962), were performed at the Department of Clinical Chemistry, Royal Manchester Children's Hospital, and the results were interpreted by reference to the normal ranges established in that laboratory by 95% confidence limits.

### Results

The age, stage, DBH levels, urinary catecholamine excretion and clinical status of the neuroblastoma patients are shown in Table I. Control subjects were undergoing investigations for a variety of disorders.

The serum DBH activities of the neuroblastoma patients were similar to those of age-matched controls (Table II). Studies in our laboratory have shown that DBH rises with age to approach adult values ($0–100\,iu\,l^{-1}$, mean $29\,iu\,l^{-1}$) at around 7 years, with no difference between the sexes.

**Table I** Urinary excretion of catecholamines and metabolites ($\mu g\,mg^{-1}$ creatinine) in neuroblastoma patients

| *Case* | *Age (years)* | *Stage* | *DBH $IU\,l^{-1}$* | *Total Catecholamines (as Dopamine)* | *Total metadrenalines* | *HMMA* | *Clinical Status* | *Course* |
|---|---|---|---|---|---|---|---|---|
| 1 | 0.1 | I | 2.1 | 1.20 | 2.80 | 8.0 | NED | Died 1.7 yr. Intestinal obstruction |
| 2 | 0.2 | IVS | 66.3 | 9.30↑ | 190.00↑ | 447.0↑ | Disease | Died 0.8 yr. Bleeding oesophageal varices. Residual disease |
| 3 | 0.7 | IV | 2.7 | 9.60↑ | 1.20 | 15.0 | Disease | Died 1.2 yr. NBL |
| 4 | 0.7 | I | 0.4 | 2.77 | 1.14 | 9.8 | NED | NED 5 yr. |
| 5 | 0.8 | IV | 2.1 | 9.60↑ | 1.03 | 10.7 | Disease | Died 0.9 yr. NBL |
| 6 | 0.8 | IV | 2.8 | 16.10↑ | 0.90 | 9.3 | Disease | Died 1.9 yr. NBL |
| 7 | 1.0 | IV | 2.3 | — | — | — | Disease | Died 1.3 yr. NBL |
| 8 | 1.0 | IV | 9.7 | — | — | — | Disease | Died 1.8 yr. |
| | 1.3 | | — | 12.00↑ | 0.85 | 20.6↑ | Disease | Pneumocystis carinii pneumonia; residual disease |
| 9 | 1.2 | IV | 0.9 | 12.70↑ | 6.70↑ | 117.0↑ | Disease | Died 1.7 yr. NBL |
| 10 | 1.5 | IV | 2.4 | 11.20↑ | 0.77 | 5.1 | Disease | Died 1.8 yr. NBL |
| 11 | 1.8 | III | — | 3.30↑ | — | 14.4↑ | Disease | NED 7.8 yr. |
| | 2.0 | | 4.9 | 1.20 | 0.77 | 4.4 | NED | |
| 12 | 2.0 | IV | 2.4 | 9.20↑ | 2.60 | 16.6↑ | Disease | Died 2.4 yr. NBL |
| 13 | 3.0 | IV | 11.9 | 2.10 | 10.30↑ | 32.4↑ | Disease | Died 3.4 yr. NBL |
| 14 | 3.7 | IV | 9.7 | 0.31 | 5.20↑ | 52.90↑ | Disease | Died 4.4 yr. NBL |
| 15 | 3.7 | IV | 7.2 | 18.80↑ | 23.00↑ | 22.00↑ | Disease | Died 3.8 yr. NBL |
| 16 | 4.0 | III | 3.0 | 1.60 | 0.48 | 5.90 | NED | NED 7.8 yr. |
| 17 | 4.2 | IV | 7.7 | 14.60↑ | 1.60 | 7.70 | Disease | Died 4.3 yr. NBL |
| 18 | 4.2 | IV | 7.3 | 2.39↑ | 3.90↑ | 92.00↑ | Disease | Died 4.8 yr. NBL |
| 19 | 4.4 | IV | 7.9 | 20.30↑ | 7.30↑ | 130.00↑ | Disease | Died 5.8 yr. NBL |
| 20 | 4.4 | III | 10.4 | 17.40↑ | 3.70↑ | 9.50↑ | Disease | Died 5.6 yr. NBL |
| 21 | 4.4 | IV | 9.8 | 15.00↑ | 14.40↑ | 68.30↑ | Disease | Died 5.4 yr. NBL |
| 22 | 4.8 | I.V | 12.0 | 3.45↑ | 1.60 | 5.70 | Disease | Died 5.8 yr. NBL |
| 23 | 5.0 | III | 15.1 | 12.90↑ | 1.70 | 4.70 | Disease | Died 5 yr. NBL |
| 24 | 5.0 | IV | 6.6 | 26.30↑ | 0.72 | 11.20↑ | Disease | Died 6.2 yr. NBL |
| 25 | 5.5 | IV | 0.3 | 0.67 | 0.65 | 6.75 | NED | Died 5.8 yr. NBL |
| 26 | 7.2 | IV | 19.4 | 1.10 | 1.06 | 12.40↑ | Disease | Died 8.7 yr. NBl |

NED = No evidence of disease.
NBL = Neuroblastoma.
↑ = Level above 95% confidence limits of controls.

**Table II** Serum DBH levels (iu $l^{-1}$)

| Age (years) | Controls No. | Controls Range | Controls Mean | Neuroblastoma Patients with disease No. | Range | Mean | Neuroblastoma Patients without disease No. | Range | Mean |
|---|---|---|---|---|---|---|---|---|---|
| <2 | 17 | 0–12.7 | 3.12 | 8 | 0.9–9.7 | 4.0 | 2 | 0.4–2.1 | 1.25 |
| 2–4 | 12 | 2.7–18.5 | 12.9 | 4 | 7.2–11.9 | 9.6 | 2 | 3.0–4.9 | 3.95 |
| 4–6 | 12 | 6.4–29.3 | 14.7 | 8 | 6.6–15.1 | 9.5 | 1 | 0.2 | |
| 6–9 | 17 | 4.0–35.0 | 17.0 | 1 | 19.4 | | 0 | | |
| | 58 | | | 21 | | | 5 | | |

Regression analysis of DBH activities of controls and neuroblastoma patients showed progressive elevation with age (Figure 2) but no significant difference between the two groups by analysis of covariance ($P = > 0.5$). Similarly, a successfully treated neuroblastoma patient (case 4) was noted to have increasing serum DBH activity as she was followed from the age of 9 months to 3.7 years (Table III).

Five control subjects had high DBH levels (Figure 2). Acute stress may explain this (Weinshilboum, 1978) as these patients were hospitalised for suspected intestinal obstruction, pneumonia, convulsions, parental abuse and investigation of ovarian teratoma.

Serial determinations of serum enzyme levels were obtained for 8 neuroblastoma patients extending over periods of 4 months to 3 years (Table III). No changes in enzyme levels were noted in relation to disease activity, nor to changes in urinary catecholamine excretion, which corresponded to disease status. However, DBH activity was affected by external factors. For example, in a child with neuroblastoma (Case 6), DBH levels increased transiently on the day after partial removal of the tumour and rose again at the age of 20 months. We attributed the postoperative elevation to transfusion with adult blood containing higher DBH levels. Pooled plasma from 5 units of whole blood had a mean DBH activity of $45.1 \pm 22.3$ iu $l^{-1}$ (range 14.7–66.2). Children receiving blood transfusions for other disorders also showed transient serum DBH elevations. The later rise in this patient was considered a normal age-related manifestation associated with increased sympathetic activity as the child began to walk. The changes in DBH activity seem to be unrelated to the amount of residual tumour and the urinary catecholamine output. In another patient (Case 26) DBH levels were measured repeatedly from diagnosis to death and at no time were they above those of the age-matched control group; the DBH levels bore no relationship to the tumour size or urinary catecholamine output and fell prior to the child's death with disseminated disease. Another patient (Case 2) had a transient elevation in DBH (66.3 iu $l^{-1}$ compared with the control range of 0–12.2 iu $l^{-1}$ after a blood transfusion.

### Discussion

The interpretation of serum DBH levels in children is more difficult than in adults, in whom DBH activity is not age-dependent and values are generally higher. At birth serum DBH levels are almost undetectable, but they rise during the first years of life, and they reach adult values at 7–8 years (Weinshilboum, 1978). Evaluation of serum DBH levels thus requires comparison with control subjects of the same age group, measured by the same assay.

Neuroblastoma cells, both human (De Potter *et al.*, 1974; Biedler *et al.*, 1978) and mouse (Anagnoste *et al.*, 1972) contain DBH and catecholamines. In phaeochromocytoma patients, elevated serum DBH and urinary catecholamines fall after removal of the tumour (Aunis & Bouclier, 1977). Neuroblastoma is also associated with increased catecholamine excretion. Serum DBH levels might thus be expected to rise in these patients, too, especially in those whose tumours secrete predominantly noradrenalines. Of our 21 neuroblastoma patients with active disease, one excreted excess of HMMA only, 7 excreted excess amounts of total catecholamines only, and 13 excreted 2 or more metabolites in excess. All patients had DBH levels similar to age-matched controls. The lack of correlation between urinary metadrenaline excretion and serum DBH may be due to biochemical differences of neuroblastoma cells. In sympathetically innervated tissue 2 types of

**Figure 2** Serum DBH of control (▽) and neuroblastoma (●) patients. C = Control P = Patients.

**Table III** Serial serum DBH and urinary chatecholamines and metabolites ($\mu g\,mg^{-1}$ creatinine) on neuroblastoma patients

| *Case* | *Age (years)* | *Stage* | *DBH IU l^-1* | *Total Catecholamines (as Dopamine)* | *Total Metadrenalines* | *HMMA* | *Clinical Status* |
|---|---|---|---|---|---|---|---|
| 4 | 0.8 | I | 0.4 | 2.77 | 1.14 | 9.80 | NED |
| | 3.0 | | 7.5 | 1.00 | 0.69 | 11.80 | NED: walking |
| | 3.1 | | 10.4 | 0.21 | 0.91 | 9.20 | NED |
| | 3.2 | | 10.3 | 0.83 | 0.79 | 11.80 | NED |
| | 3.4 | | 10.5 | 1.10 | 0.69 | 11.10 | NED |
| | 3.7 | | 13.8 | 1.33 | 1.10 | 10.80 | NED |
| 6 | 0.8 | IV | 2.8 | 16.10↑ | 0.90 | 9.30 | Disease |
| | 0.8 | | 13.1 | — | — | — | Postoperative transfusion |
| | 1.0 | | 3.7 | — | — | — | Disease |
| | 1.2 | | 4.8 | — | — | — | Disease |
| | 1.5 | | 4.7 | 16.70↑ | 1.10 | 7.96 | Disease |
| | 1.6 | | 9.2 | — | — | — | Disease: walking |
| | 1.7 | | 12.4 | 6.2 | 1.60 | 6.70 | Disease |
| | 1.8 | | 12.0 | 7.2 | 0.82 | 9.60 | Disease |
| | 1.9 | | — | — | — | — | Died: neuroblastoma |
| 9 | 1.2 | IV | 0.90 | 12.70↑ | 6.70↑ | 117.00↑ | Disease |
| | 1.4 | | 1.02 | 35.80↑ | 5.60↑ | 143.00↑ | Disease |
| | 1.6 | | — | 56.00↑ | 3.70↑ | 146.00↑ | Disease |
| | 1.7 | | — | — | — | — | Died: neuroblastoma |
| 12 | 1.8 | III | — | 3.30↑ | — | 14.40↑ | Disease |
| | 2.0 | | 4.90 | 1.20 | 0.77 | 4.40 | NED |
| | 3.0 | | 14.60 | 0.63 | — | 6.70 | NED |
| | 7.8 | | — | — | — | — | Alive: NED |
| 16 | 4.0 | III | 3.0 | 1.60 | 0.48 | 5.90 | ? Disease |
| | 5.0 | | 6.7 | 0.31 | 0.16 | 2.70 | NED |
| | 5.7 | | 8.1 | 0.58 | 0.68 | 6.00 | NED |
| | 6.0 | | 6.9 | 0.78 | 0.58 | 5.30 | NED |
| | 7.7 | | — | — | — | — | Alive: NED |
| 21 | 4.4 | IV | 9.8 | 15.00↑ | 14.40↑ | 68.30↑ | Disease |
| | 4.7 | | 23.1 | — | — | — | Disease |
| | 4.8 | | 23.4 | — | — | — | Disease |
| | 5.3 | | 26.7 | — | — | — | Disease |
| | 5.4 | | — | — | — | — | Died: neuroblastoma |
| 25 | 5.4 | IV | 0.2 | — | — | — | NED |
| | 5.5 | | 0.3 | 0.67 | 0.65 | 6.75 | NED |
| | 5.6 | | 0.5 | 0.80 | 0.41 | 8.20 | NED |
| | 5.7 | | 1.2 | — | — | — | Disease |
| | 5.8 | | — | — | — | — | Died: neuroblastoma |
| 26 | 7.3 | IV | 19.4 | 1.10 | 1.06 | 12.40↑ | Disease |
| | 7.7 | | 19.2 | 0.56 | 0.49 | 4.40 | NED |
| | 7.8 | | 25.4 | 0.52 | 0.63 | 5.00 | NED |
| | 8.1 | | 27.5 | — | — | — | NED |
| | 8.2 | | 27.7 | 1.32 | 0.83 | 5.50 | NED |
| | 8.3 | | 26.5 | — | — | — | NED |
| | 8.6 | | 22.1 | 5.60↑ | 8.70↑ | 37.30↑ | Disease |
| | 8.7 | | — | — | — | — | Died: neuroblastoma |

noradrenaline storage vesicles have been identified. Only one of these contains DBH, the other possessing little, if any, DBH activity. Neuroblastoma cells lack enzyme-containing vesicles (De Potter *et al.*, 1974; 1978*a*). Studies of subcellular distribution of catecholamines and enzymes have shown that in both human (De Potter *et al.*, 1974) and mouse (De Potter *et al.*, 1978*b*; 1980) neuroblastoma, most of the DBH is associated with the plasma membrane. This bound DBH is not released by exocytosis.

In conclusion, our study has demonstrated that any changes in serum DBH level observed in neuroblastoma patients could be accounted for by increasing age and physical activity or by blood transfusion with adult blood containing higher DBH activity than present in the patient. We found no correlation with disease status or urinary catecholamine excretion.

We thank the National Cancer Institute of Cairo University and the Research Endowment Fund of the Central Birmingham Health District for financial support.

### References

ANAGNOSTE, B., FREEDMAN, L.S., GOLDSTEIN M., BROOM, J. & FUXE, K. (1972). Dopamine-β-hydroxylase activity in mouse neuroblastoma tumours and in cell culture. *Proc. Natl Acad. Sci.*, **69**, 1883.

AUNIS, D. & BOUCLIER, M. (1977). Comparative study of plasma dopamine-beta-hydroxylase activities in noradrenaline-secreting and adrenaline-secreting phaeochromocytomae. *Clin. Exp. Pharmacol. Physiol.*, **4**, 359.

BIEDLER, J.L., ROFFLER-TARLOV, S., SCHACHNER, M. & FREEDMAN, L.S. (1978). Multiple neurotransmitter synthesis by human neuroblastoma cell lines and clones. *Cancer Res.*, **38**, 3751.

BREWSTER, M.A. & BERRY, D.H. (1979). Serial studies of serum dopamine-β-hydroxylase and urinary vanillylmandelic and homovanillic acids in neuroblastoma. *Med. Pediatr. Oncol.*, **6**, 93.

DE POTTER, W.P., DE SCHAEPDRYVER, A.F., DESMET, F., DELBEKE, M.J. & HOOFT, C. (1974). Subcellular distribution of catecholamines and enzymes in human neuroblastoma. *Experientia*, **30**, 1323.

DE POTTER, W.P., FRAEYMAN, N.H. PALM, J.W. & DESCHAEPDRYVER, A.F. (1978*a*). Localization of noradrenaline and dopamine-beta-hydroxylase in C1300 mouse neuroblastoma. A biochemical and electronmicroscopic study. *Life Sci.*, **23**, 2665.

DE POTTER, W.P., FRAEYMAN, N.H., PLUM, J. & DESCHAEPDRYVER, A.F. (1978*b*). Immunohistochemical evidence for a plasma membrane localization of dopamine-beta-hydroxylase in C1300 mouse neuroblastoma. *Arch. Int. Physiol Biochim.*, **86**, 424.

DE POTTER, W.P., FRAEYMAN, N.H., PLUM, J. & DESCHAEPDRYVER, A.F. (1980). Immunohistochemical evidence for a plasma membrane localization of dopamine-beta-hydroxylase in the mouse neuroblastoma. *Tissue Cell*, **12**, 227.

EVANS, A.E., D'ANGIO, G.J. & RANDOLPH, J. (1971). A proposed staging for children with neuroblastoma. *Cancer*, **27**, 374.

GITLOW, S.E., DZIEDZIC, L.B., STRAUSS, L., GREENWOOD, S.M. & DZIEDZIC S.W. (1973). Biochemical and histologic determinants in the prognosis of neuroblastoma. *Cancer*, **32**, 898.

GOLDSTEIN, M., FREEDMAN, L.S., BOHOUN, A.C. & GUERINOT, F. (1972). Serum dopamine-β-hydroxylase activity in neuroblastoma. *N. Engl. J. Med.*, **286**, 1123.

KATO, T., KUZUYA, H. & NAGATSU, T. (1974). A simple and sensitive assay for dopamine-beta-hydroxylase activity by dual-wavelength spectrophotometry. *Biochem. Med.*, **10**, 320.

LAUG, W.E., SIEGEL, S.E., SHAW, K.N.F., LANDING, B., BAPTISTA, J. & GUTENSTEIN, M. (1978). Initial urinary catecholamine metabolite concentrations and prognosis in neuroblastoma. *Pediatrics*, **62**, 77.

NAGATSU, T. & UDENFRIEND, S. (1972). Photometric assay of dopamine-β-hydroxylase activity in human blood. *Clin. Chem.*, **18**, 980.

PISANO, J.J. (1960). A simple analysis for normetanephrine and metanephrine in urine. *Clin. Chim. Acta*, **5**, 406.

PISANO, J.J., CROUT, J.R. & ABRAHAM, D. (1962). Determination of 3-methoxy-4-hydroxymandelic acid in urine. *Clin. Chim. Acta*, **7**, 285.

ROCKSON, S.G., STONE, R.A., ODERE, F. & GUNNELLS, J.C. (1976). Plasma dopamine-β-hydroxylase in the diagnosis of neuroblastoma. *Cancer*, **37**, 386.

VARLEY, H. (1967). *Practical Clinical Chemistry*. London: Heinemann. p. 684.

WEINSHILBOUM, R.M. (1978). Serum dopamine-β-hydroxylase. *Pharmacol. Rev.*, **30**, 133.

WEINSHILBOUM, R. & AXELROD, J. (1971). Serum dopamine-β-hydroxylase: Decrease after chemical sympathectomy. *Science*, **173**, 931.

WEINSHILBOUM, R.M., THOA, N.B., JOHNSON, D.G., KOPIN, I.J. & AXELROD J. (1971). Proportional release of norepinephrine and dopamine-β-hydroxylase from sympathetic nerves. *Science*, **174**, 1349.